# Identification of TIMP1 as an inflammatory biomarker associated with temporal lobe epilepsy based on integrated bioinformatics and experimental analyses

**DOI:** 10.1186/s12974-023-02837-3

**Published:** 2023-06-26

**Authors:** Ya He, Hongxia Zhang, Limin Ma, Jingang Li, Fei Wang, Hui Zhou, Guangliang Zhang, Yuetao Wen

**Affiliations:** 1 Department of Physical Examination Center, Chongqing University Jiangjin Hospital, Chongqing University, Chongqing, China; 2 Department of Neurosurgery, Yongchuan Hospital of Chongqing Medical University, Chongqing, China; 3 Department of Neurology, The Second Affiliated Hospital of Chongqing Medical University, Chongqing, China; 4 Department of Neurology, Chongqing University Three Gorges Hospital, Chongqing University, Chongqing, China; 5 Department of Neurosurgery, Chongqing University Jiangjin Hospital, Chongqing University, Chongqing, China

**Keywords:** Temporal lobe epilepsy, Inflammation-related genes, Bioinformatics analysis, TIMP1, Biomarker

## Abstract

**Background:**

Epilepsy is the second most prevalent neurological disease. Although there are many antiseizure drugs, approximately 30% of cases are refractory to treatment. Temporal lobe epilepsy (TLE) is the most common epilepsy subtype, and previous studies have reported that hippocampal inflammation is an important mechanism associated with the occurrence and development of TLE. However, the inflammatory biomarkers associated with TLE are not well defined.

**Methods:**

In our study, we merged human hippocampus datasets (GSE48350 and GSE63808) through batch correction and generally verified the diagnostic roles of inflammation-related genes (IRGs) and subtype classification according to IRGs in epilepsy through differential expression, random forest, support vector machine, nomogram, subtype classification, enrichment, protein‒protein interaction, immune cell infiltration, and immune function analyses. Finally, we detected the location and expression of inhibitor of metalloproteinase-1 (TIMP1) in epileptic patients and kainic acid-induced epileptic mice.

**Results:**

According to the bioinformatics analysis, we identified TIMP1 as the most significant IRG associated with TLE, and we found that TIMP1 was mainly located in cortical neurons and scantly expressed in cortical gliocytes by immunofluorescence staining. We detected decreased expression of TIMP1 by quantitative real-time polymerase chain reaction and western blotting.

**Conclusion:**

TIMP1, the most significant IRG associated with TLE, might be a novel and promising biomarker to study the mechanism of epilepsy and guide the discovery of new drugs for its treatment.

**Supplementary Information:**

The online version contains supplementary material available at 10.1186/s12974-023-02837-3.

## Background

Epilepsy, the second most prevalent neurological disease, is caused by abnormalities and excessive or highly synchronized electrical activity of neurons in the brain [1]. Worldwide, more than 65 million people are estimated to have epilepsy. In high-income countries, there are 5–8 epilepsy patients per 1000 people, while in low-income countries, there are 10 epilepsy patients per 1000 people [2]. Temporal lobe epilepsy (TLE) is the most common subtype of epilepsy, accounting for 60% of epilepsy cases, and it originates in the hippocampus or amygdala [3]. This condition may induce severe neurobehavioral comorbidities, such as cognitive decline, anxiety, depression, schizophrenia, and autism [4]. Although there are many antiseizure drugs, approximately 30% of cases are refractory to treatment, characterized by multiple seizure types and progressive cognitive and developmental deterioration [5]. Therefore, finding new therapeutic targets for epilepsy is essential.

The basic pathophysiology of epilepsy is still not fully understood, but inflammation is considered to play a key role in the occurrence and development of epilepsy [6]. Proinflammatory factors in the brain, such as interleukin-1β (Il-1β), Il-6, and tumor necrosis factor (TNF-α), initiate downstream inflammatory cascades and lead to impairment of neuronal function, which promotes the occurrence and development of epilepsy [7, 8]. For example, many inflammatory factors affect neuronal excitability by regulating the function of neuronal receptors. Accumulated evidence has shown that the *N*-methyl-d-aspartic acid (NMDA) receptor is coexpressed with IL-1R1, one receptor of IL-1β, on hippocampal pyramidal neurons, and IL-1β-mediated activation of neuronal IL-1R1 induces Src kinase-mediated tyrosine phosphorylation of the NR2B subunit of the NMDA receptor, resulting in Ca^2+^ influx into neurons and neuronal excitotoxicity [9, 10]. IL-1β can also inhibit GABA-mediated Cl^−^ fluxes and reduce inhibitory transmission as well as increase neuronal glutamate release via the activation of inducible nitric oxide synthase in astrocytes [9, 10]. TNF-α activates the recruitment of AMPA receptors lacking the GluR2 subunit at neuronal membranes and contributes to the formation of molecular conformations that benefit Ca^2+^ influx into neurons and induce endocytosis of GABA_A_ receptors [11]. Moreover, neuroinflammation increases blood brain barrier (BBB) permeability, leading to the invasion of the brain by proinflammatory cytokines, which aggravates the neuronal damage caused by inflammation and plays a key role in seizure generation and exacerbation [4]. Therefore, neuroinflammation is likely closely associated with the occurrence and development of epilepsy, and inflammation might be a target for epilepsy therapy [12]. However, the potential mechanisms of neuroinflammation in epilepsy remain unknown, and which of the inflammation-related genes (IRGs) are strongly associated with epilepsy has yet to be determined. Therefore, further studies on the identification of inflammatory biomarkers associated with epilepsy are urgently needed, which can help provide new molecular targets for studying the mechanism of epilepsy and discovering new drugs for treatment.

In our study, we merged the GSE48350 and GSE63808 datasets through batch correction and generally verified the diagnostic roles of IRGs and subtype classification according to IRGs in epilepsy. Based on differential expression, random forest (RF), support vector machine (SVM), nomogram, subtype classification, enrichment, protein‒protein interaction (PPI), immune cell infiltration, and immune function analyses, we identified inhibitor of metalloproteinase-1 (TIMP1) as the most significant IRG associated with temporal lobe epilepsy (TLE). Finally, we detected the expression of TIMP1 in epileptic patients and mice.

## Materials and methods

### Obtaining and preprocessing the datasets

TLE usually originates in the hippocampus. Thus, hippocampal datasets, GSE48350 and GSE63808, were downloaded from the GEO database (http://www.ncbi.nlm.nih.gov/geo/). The GSE48350 dataset contains 43 hippocampal specimens from control cases, and the GSE63808 dataset contains 129 hippocampal specimens from patients with TLE. We obtained a new dataset after the merging control cases and TLE cases through batch corrections by using the R packages “limma” and “sva”. Two hundred IRGs were acquired from hallmark gene sets of gene set enrichment analysis (GSEA). We extracted the expression of those 200 IRGs in the new dataset and identified differentially expressed IRGs in TLE compared with controls.

### RF, SVM and nmomogram analyses

To further verify the diagnostic capacity of differentially expressed IRGs in TLE, the RF and SVM models were constructed by using the R packages “randomForest” and “e1071”, respectively. RF, as a machine learning algorithm based on decision-tree theory for solving classification problems, has a high degree of predictive accuracy by using bootstrap aggregation and randomization of predictors [13]. SVM is a powerful classification tool that has the function of maximizing (support) the separating margin (vector) [14]. These two machine methods were used to identify significant predictive genes of differentially expressed IRGs in TLE. All parameters in RF and SVM were automatically adjusted using the R package “caret”, and five-fold cross-validation was performed to assess each model. The R package “DALEX” was used to generate the residual distribution and feature importance results among them. The area under the receiver operating characteristic (ROC) curve (AUC) was visualized using the R package “pROC”. The AUC was used to judge the accuracy and efficiency of the predictive model, where a higher AUC represents a model with higher accuracy [15]. The top five most important variables were considered the key predictive genes associated with TLE in the RF analysis. Based on RF and SVM analyses, we identified core IRGs from differentially expressed IRGs. Then, we used the core IRGs from RF and SVM to perform nomogram analysis by using the R package “RMS” to predict the occurrence of TLE [16]. A calibration curve was drawn to assess the predictive accuracy of the nomogram. Moreover, the decision curve and clinical impact curve were plotted to evaluate the clinical value of the nomogram.

### Subtype classification analyses

To further verify the important roles of IRGs in TLE, TLE patients were divided into clusters based on differentially expressed IRGs through consensus clustering by using the R package “ConsensusClusterPlus”, which were named IRGcluster A, B, C, etc. [17]. Similarly, TLE patients were divided into clusters according to differentially expressed genes (DEGs) obtained from differential expression analysis between IRGcluster groups with |logFC| > 0.585 and adjusted *P* < 0.05, which were named genecluster A, B, C, etc. Moreover, principal component analysis (PCA) was conducted to reduce the dimensionality and evaluate the independence of each subgroup.

### PPI and enrichment analyses

Based on the differential expression analysis between IRGcluster groups, we obtained DEGs, and PPI analysis of these genes was performed using the STRING online tool (https://cn.string-db.org/cgi/input.pl). These genes also underwent Gene Ontology (GO) and Kyoto Encyclopedia of Genes and Genomes (KEGG) pathway enrichment analyses by using the R package “enrichplot”. Moreover, GSEA was employed to identify the biological functions and pathways in the IRGcluster groups by using the R package “enrichplot”.

### Immune cell infiltration and immune function

Single-sample GSEA (ssGSEA) was used to evaluate the abundances of 23 immune cell subtypes in TLE patients [18]. The correlation between differentially expressed IRGs and the 22 immune cell subtypes was evaluated using Spearman analysis. The differences in the 22 types of immune cells in the IRGcluster subgroups and genecluster subgroups were analyzed. Immune function scores were obtained by using the R packages “limma”, “GSVA” “GSEABase” and “reshape2” [19]. The correlation between differentially expressed IRGs and the immune function score was evaluated using Spearman analysis. The differences in immune function scores in the IRGcluster subgroups and genecluster subgroups were analyzed.

### Human brain tissue

Twelve temporal neocortex samples from patients with traumatic brain injury and without a history of other neurological diseases who underwent craniotomy were used for the control group, and 12 temporal neocortex samples from TLE patients were used for the TLE group. Temporal neocortex samples were collected from the Chongqing University Jiangjin Hospital and First Affiliated Hospital of Chongqing Medical University. This study was performed in accordance with the Declaration of Helsinki and the ethical principles of the National Institutes of Health and approved by the Ethics Committee of the Chongqing University Jiangjin Hospital and Second Affiliated Hospital of Chongqing Medical University. All patients and their guardians were informed of the use of brain tissues and provided informed consent. A portion of each specimen was cut into 16 μm frozen sections for immunofluorescence staining, and the remaining specimen was stored at − 80 °C for quantitative real-time polymerase chain reaction (qRT‒PCR) and western blotting.

### Kainic acid (KA)-induced epilepsy

Adult male C57BL/6 mice (8–10 weeks and 20–25 g) were obtained from the Experimental Animal Center of Chongqing Medical University, and this study was approved by the Committee on Animal Research of Chongqing Medical University. Control mice and kainic acid (KA)-induced epileptic mice were treated according to the method described in a previous study [20]. The cortices and hippocampi were obtained from control and KA mice under anesthesia using the intraperitoneal injection of sodium pentobarbital (50 mg/kg). A portion of each specimen was cut into 16 μm frozen sections for immunofluorescence staining, and the remaining specimen was stored at − 80 °C for qRT‒PCR and western blotting.

### Immunofluorescence staining and western blotting

Immunofluorescence staining and western blotting were performed according to a previous study [20]. For immunofluorescence staining, the following primary antibodies were used: rabbit anti-TIMP1 (1:50, Proteintech), mouse anti-NeuN (1:50, Millipore) and mouse anti-GFAP (1:50, Abcam), and the following secondary antibodies were used: fluorescein isothiocyanate (FITC)-conjugated donkey anti-rat IgG (1:100, Proteintech) and Alexa Fluor 555-conjugated donkey anti-mouse IgG (1:100, Beyotime). For western blotting, the following primary antibodies were used: rabbit anti-TIMP1 (1:1000, Proteintech) and rabbit anti-GAPDH (1:1000, Proteintech), while horseradish peroxidase (HRP)-goat anti-rabbit immunoglobulin G (1:3000, Proteintech) was used as the secondary antibody.

### qRT‒PCR

qRT‒PCR was used to analyze the TIMP1 mRNA level in TLE patients and KA mice according to the method described in a previous study [21]. GAPDH expression served as an internal control. The primer sequences for patients are as follows: *TIMP1* (forward: TTCCAGTCCCGTCACCTT; reverse: CAGGCTTCAGCTTCCACTC) and *GAPDH* (forward: CAGGAGGCATTGCTGATGAT; reverse: GAAGGCTGGGGCTCATTT). The primer sequences for mice are as follows: *TIMP1* (forward: TCACTGTTTGTGGACGGA; reverse: AGGCTTCAGGTCATCGG) and *GAPDH* (forward: GGTTGTCTCCTGCGACTTCA; reverse: TGGTCCAGGGTTTCTTACTCC).

### Statistical analysis

GraphPad Prism (version 6.07) and R software (version 4.1.3) were applied for data processing, statistical analysis, and graph visualization. The correlation analysis was performed using the Spearman test, and the Wilcoxon test was performed to compare the differences between different groups in datasets. The qRT‒PCR and western blotting data are presented as the mean ± standard deviation, and a t test was used to analyze and compare the differences between the two groups. Unless stated otherwise, two-tailed *P* < 0.05 was considered significant.

## Results

### Identification of differentially expressed IRGs in TLE

**Fig. 1:**
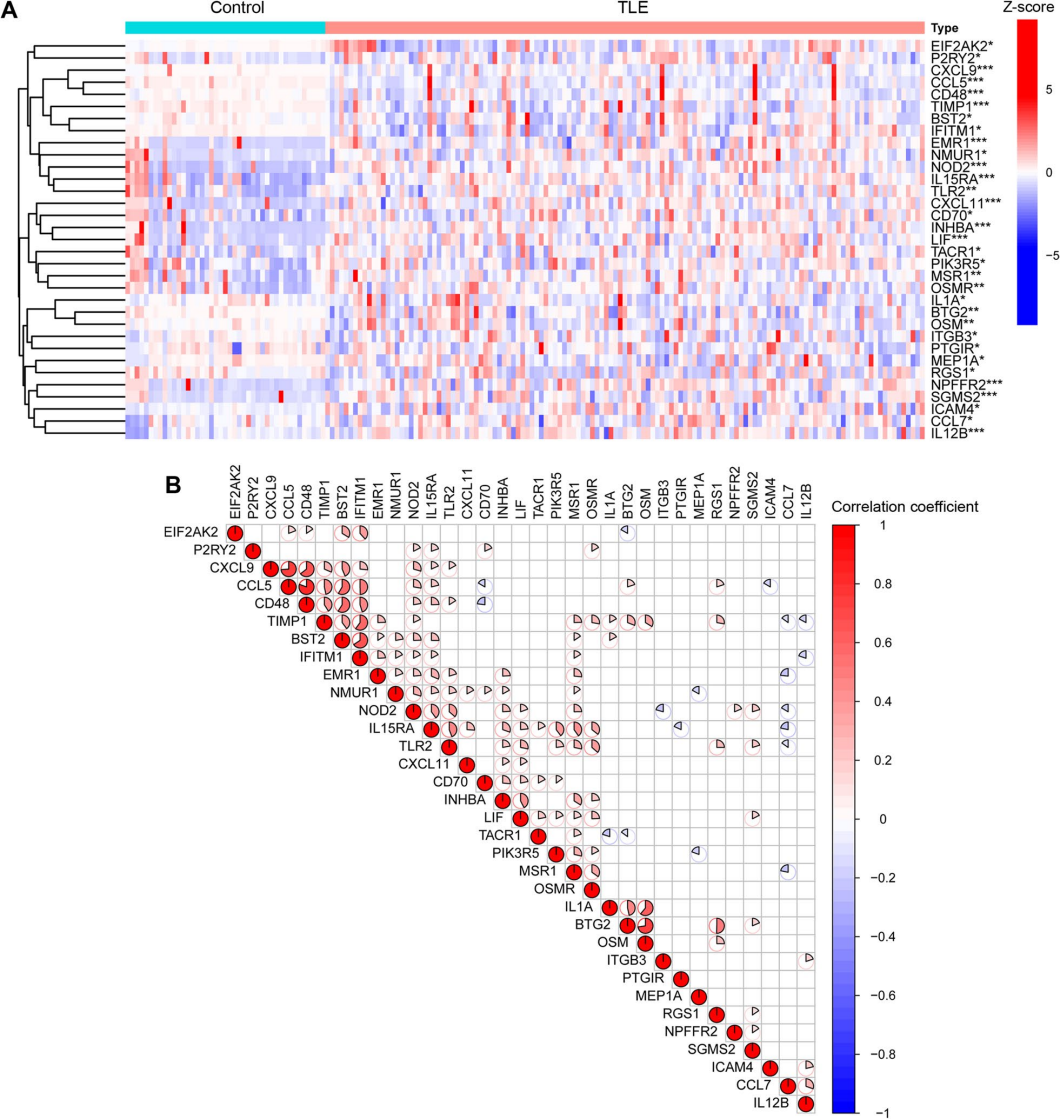
Identification of differentially expressed IRGs in the TLE. **A** Expression heat map of the 33 differentially expressed IRGs. **B** Correlation between the 33 differentially expressed IRGs. *p < 0.05, **p < 0.01, ***p < 0.001

After merging the GSE48350 and GSE63808 datasets through batch correction, we obtained a new dataset containing 43 hippocampal specimens from control cases and 129 hippocampal specimens from TLE cases. We analyzed the differential expression of 200 IRGs between the control group and TLE group by the Wilcoxon test, and we identified 33 differentially expressed IRGs (EIF2AK2, P2RY2, CXCL9, CCL5, CD48, TIMP1, BST2, IFITM1, EMR1, NMUR1, NOD2, IL15RA, TLR2, CXCL11, CD70, INHBA, LIF, TACR1, PIK3R5, MSR1, OSMR, IL1A, BTG2, OSM, ITGB3, PTGIR, MEP1A, RGS1, NPFFR2, SGMS2, ICAM4, CCL7 and IL12B), which are shown in the heatmap (Fig. 1A). Then, we used the Spearman test to analyze the correlation among these 33 differentially expressed IRGs and found some correlations among them (Fig. 1B).

### Establishment of the RF and SVM models

**Fig. 2:**
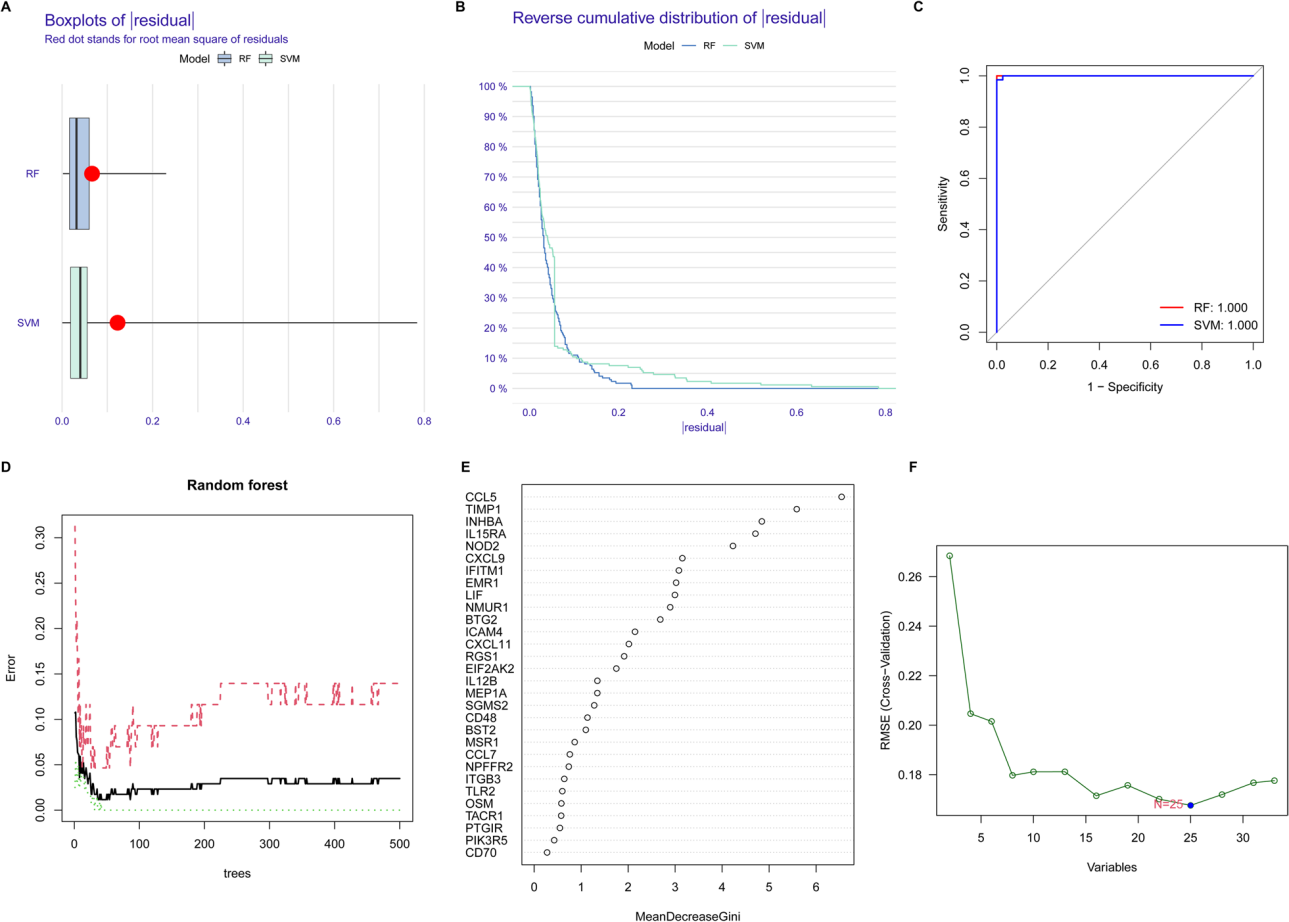
Establishment of the RF and SVM models. **A** Boxplots of residual for RF and SVM. **B** Reverse cumulative distribution of residual for RF and SVM. **C** ROC curves for RF and SVM. **D** The error of RF. **E** Mean decreasegini of differentially expressed IRGs. **F** RMSE of SVM

We established RF and SVM models based on 33 differentially expressed IRGs and found that both the RF and SVM models had minimal residuals according to the residual boxplots (Fig. 2A) and reverse cumulative distribution of residuals (Fig. 2B). Moreover, the AUC value of both the RF and SVM models was 1 (Fig. 2C). These results indicated that both the RF and SVM models had high diagnostic capacity for TLE. The RF model had low error in diagnosing TLE (Fig. 2D). The mean decrease in the Gini index reflects the effect of each variable on the heterogeneity at each node of the classification tree to compare the importance of the variables. The larger the Gini index value, the more important the variable is. As shown in Fig. 2E, the top 5 most important genes were CCL5, TIMP1, INHBA, IL15RA and NOD2, which were deemed candidate genes. The root mean square error (RMSE) is often used as a standard to measure the prediction capacity of machine learning models. As shown in Fig. 2F, the SVM model had minimum RMSE when the gene number was 25, and those 25 genes (TIMP1, CCL5, INHBA, NOD2, IL15RA, IFITM1, CXCL9, CXCL11, CD48, EMR1, EIF2AK2, BTG2, NPFFR2, MEP1A, LIF, ICAM4, OSM, SGMS2, TLR2, IL12B, NMUR1, RGS1, MSR1, BST2 and CCL7) were considered candidate genes.

### Establishment of the nomogram model

**Fig. 3:**
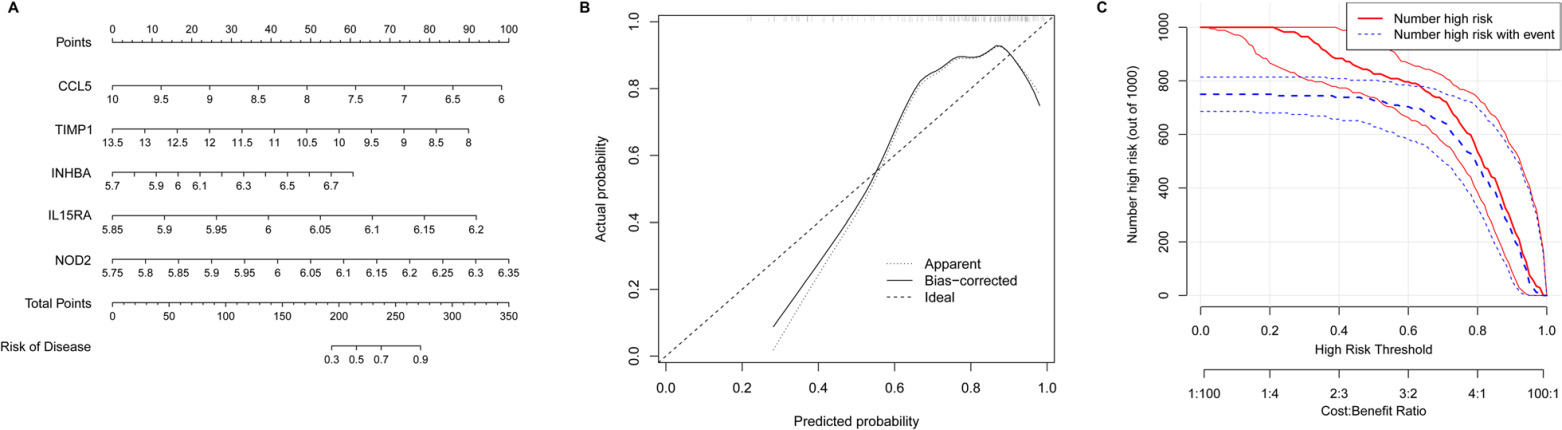
Establishment of nomogram model. **A** Nomogram model. **B** The calibration curve to assess the predictive power of the nomogram model. **C** The clinical impact curve of nomogram model

Analysis of the candidate genes by RF and SVM indicated that five IRGs (CCL5, TIMP1, INHBA, IL15RA and NOD2) were core genes. We established a nomogram model based on these five IRGs to predict the prevalence of TLE (Fig. 3A). Calibration curves showed that the diagnostic capacity of the nomogram model was accurate (Fig. 3B). The clinical impact curve indicated the high predictive capacity of the nomogram model (Fig. 3C).

### Subtype classification

**Fig. 4:**
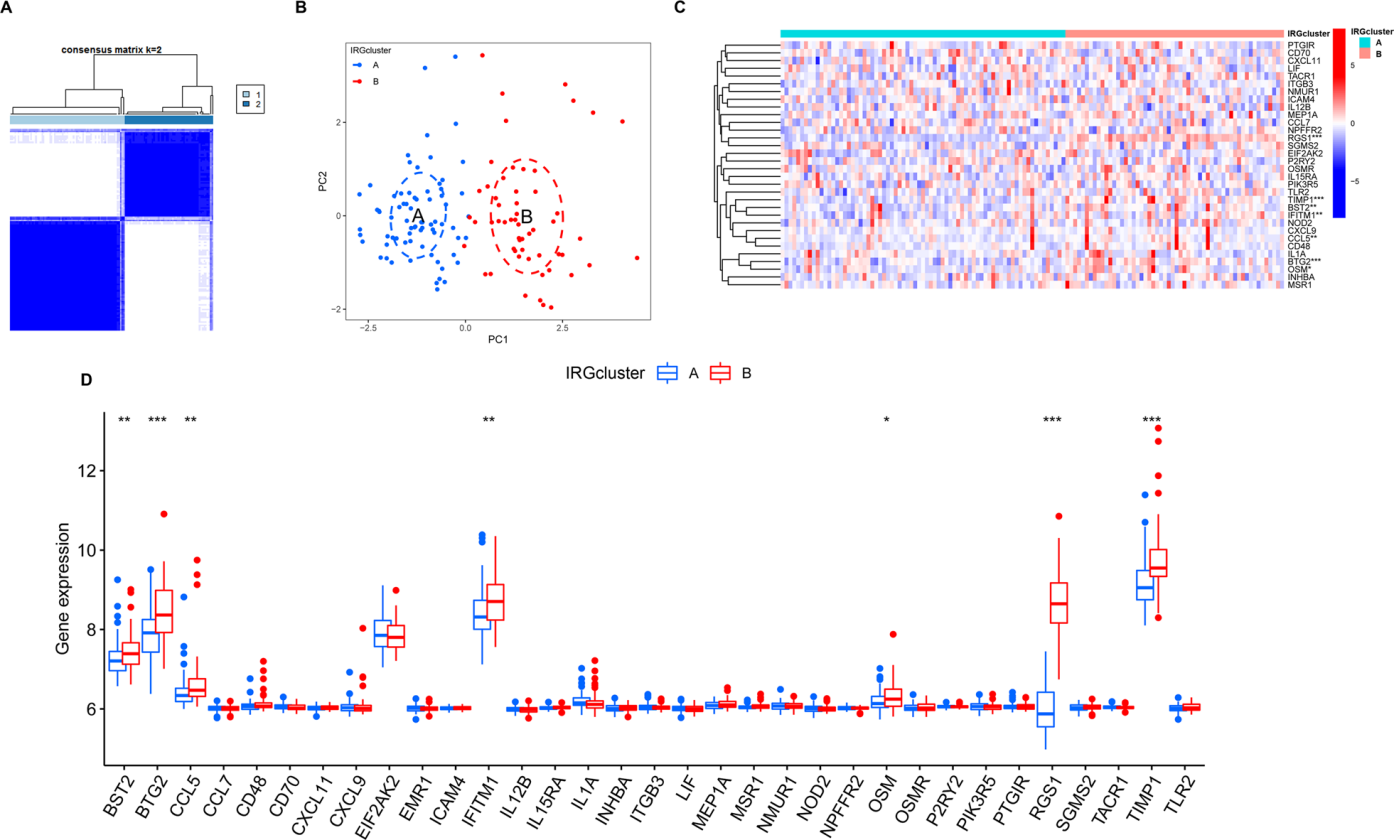
Subtype classification based on 33 differentially expressed IRGs. **A** Consensus matrices of the 33 differentially expressed IRGs for *k* = 2. **B** PCA for the expression profiles of the 33 differentially expressed IRGs. **C** Expression heat map of the 33 differentially expressed IRGs in the IRGcluster A group and IRGcluster B group. **D** Boxplot of the 33 differentially expressed IRGs in the IRGcluster A group and IRGcluster B group. *p < 0.05, **p < 0.01, ***p < 0.001

**Fig. 5:**
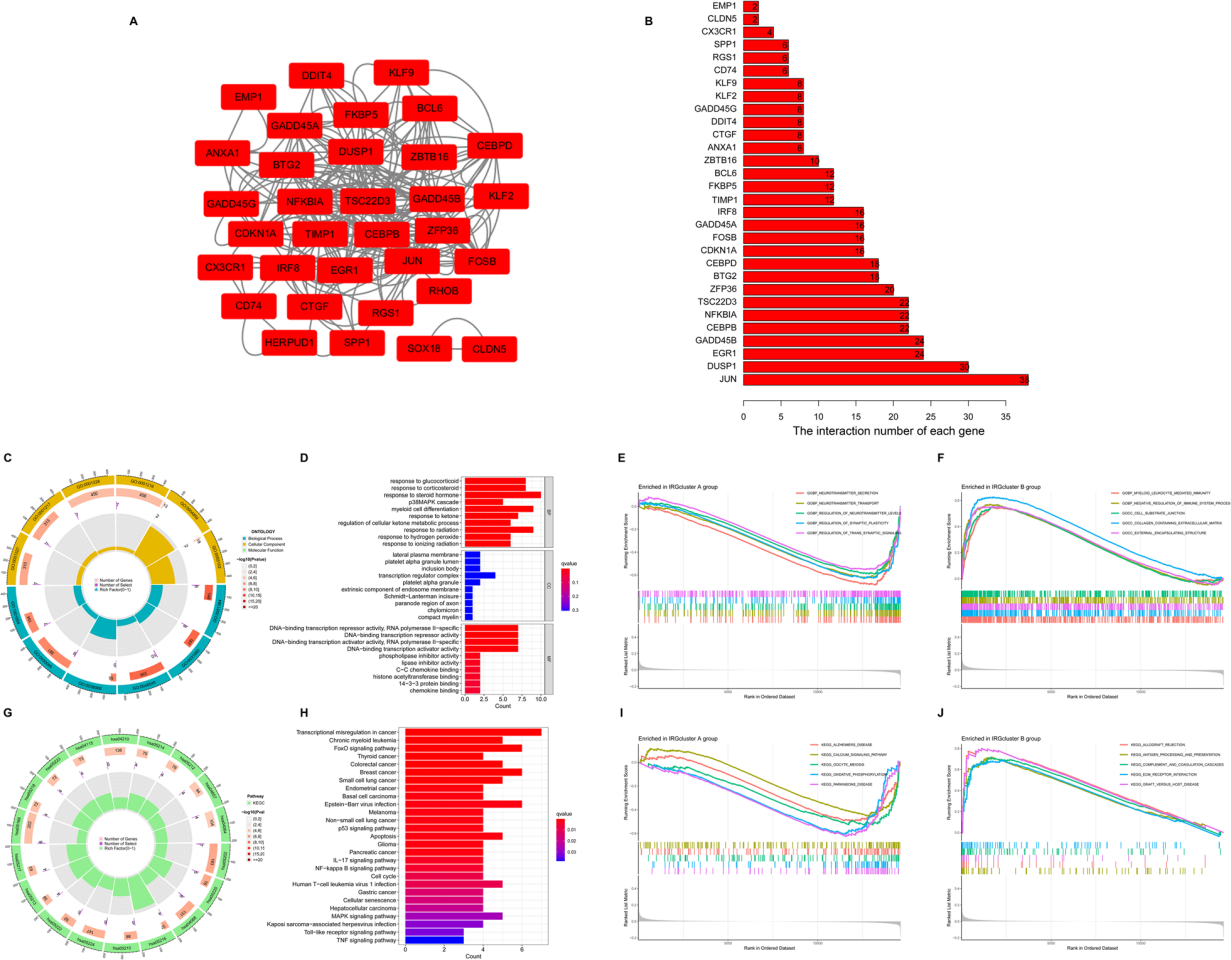
PPI and enrichment analyses. **A** PPI network of the 48 IRGs-related DEGs. **B** Top 30 edge counts of IRGs-related DEGs. **C** The circle diagram enriched in the GO analysis. **D** The top 30 significant terms of GO functional enrichment. **E** The top 5 GO pathway in the IRGcluster A group. **F** The top 5 GO pathway in the IRGcluster B group. **G** The circle diagram enriched in the KEGG analysis. **H** The top 30 significant terms of KEGG functional enrichment. **I** The top 5 KEGG pathway in the IRGcluster A group. **J** The top 5 KEGG pathway in the IRGcluster B group

Based on 33 differentially expressed IRGs, TLE patients were divided into the IRGcluster A and the IRGcluster B groups by consensus clustering (Fig. 4A), and PCA indicated that 33 differentially expressed IRGs could completely distinguish TLE patients in the IRGcluster A and IRGcluster B groups (Fig. 4B). We compared the expression of these 33 differentially expressed IRGs in the IRGcluster A and IRGcluster B groups and found that only 7 IRGs (BST2, BTG2, CCL5, ICAM4, OSM, RGS1 and TIMP1) were differentially expressed in the IRGcluster A and IRGcluster B groups (Fig. 4C, D). Comparing the expression of all genes in the IRGcluster A and B groups by using the R package “limma” under the conditions of *p* < 0.05 and |logFC| > 0.585, 48 DEGs were identified (Additional file 1: Table S1), which we named IRG-related DEGs. The 48 IRG-related DEGs were used for PPI and enrichment analyses. A PPI network among the 48 IRG-related DEGs was established using the STRING online tool and visualized using Cytoscape (version 3.7.2) (Fig. 5A), and the top 30 edge counts of IRG-related DEGs are shown in Fig. 5B. The GO terms revealed that the 48 IRG-related DEGs were significantly associated with response to glucocorticoid, platelet alpha granule lumen and DNA-binding transcription repressor activity, and RNA polymerase II-specific in the biological processes (BP), cellular components (CC) and molecular functions (MF), respectively (Fig. 5C, D). Functional annotation of GO terms was further performed using GSEA, and the top 5 pathways significantly enriched in the IRGcluster A and IRGcluster B groups are shown in Fig. 5E, F, respectively. The KEGG terms revealed that 48 IRG-related DEGs were significantly associated with transcriptional misregulation in cancer (Fig. 5G, H). Functional annotation of KEGG terms was further performed using GSEA, and the top 5 pathways significantly enriched in the IRGcluster A and IRGcluster B groups are shown in Fig. 5I, J, respectively.

**Fig. 6:**
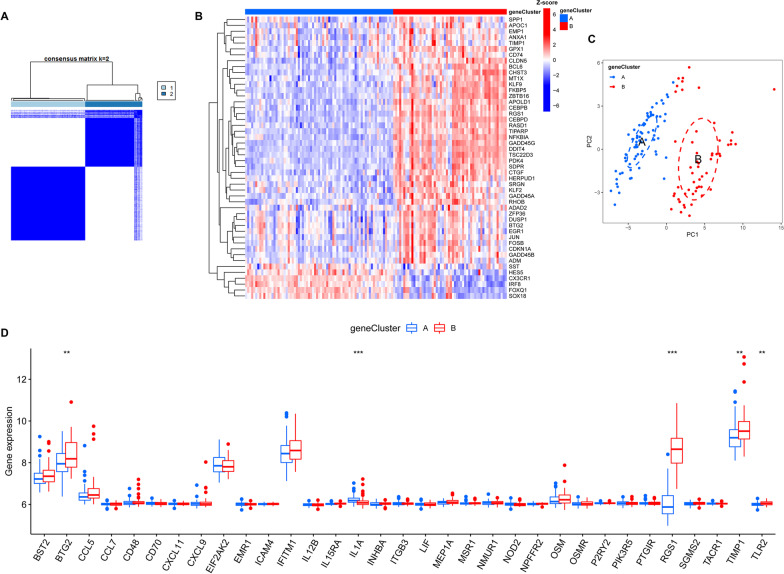
Subtype classification based on 48 IRGs-related DEGs. **A** Consensus matrices of the 48 IRGs-related DEGs for *k* = 2. **B** Expression heat map of the 48 IRGs-related DEGs. **C** PCA for the expression profiles of the 48 IRGs-related DEGs. **D** Boxplot of the 48 IRGs-related DEGs in the genecluster A group and genecluster B group. *p < 0.05, **p < 0.01, ***p < 0.001

Based on 48 IRG-related DEGs, TLE patients were divided into genecluster A and genecluster B groups by consensus clustering (Fig. 6A). We found that these 48 IRG-related DEGs were differentially expressed in the genecluster A and genecluster B groups (Fig. 6B), and PCA indicated that 48 IRG-related DEGs could completely distinguish TLE patients in the genecluster A and genecluster B groups (Fig. 6C). Moreover, we also compared the expression of 33 differentially expressed IRGs in the genecluster A and genecluster B groups and found that only 5 IRGs (BTG2, IL1A, RGS1, TIMP1 and TLR2) were differentially expressed in the genecluster A and genecluster B groups (Fig. 6D).

### Immune cell infiltration and immune function

**Fig. 7:**
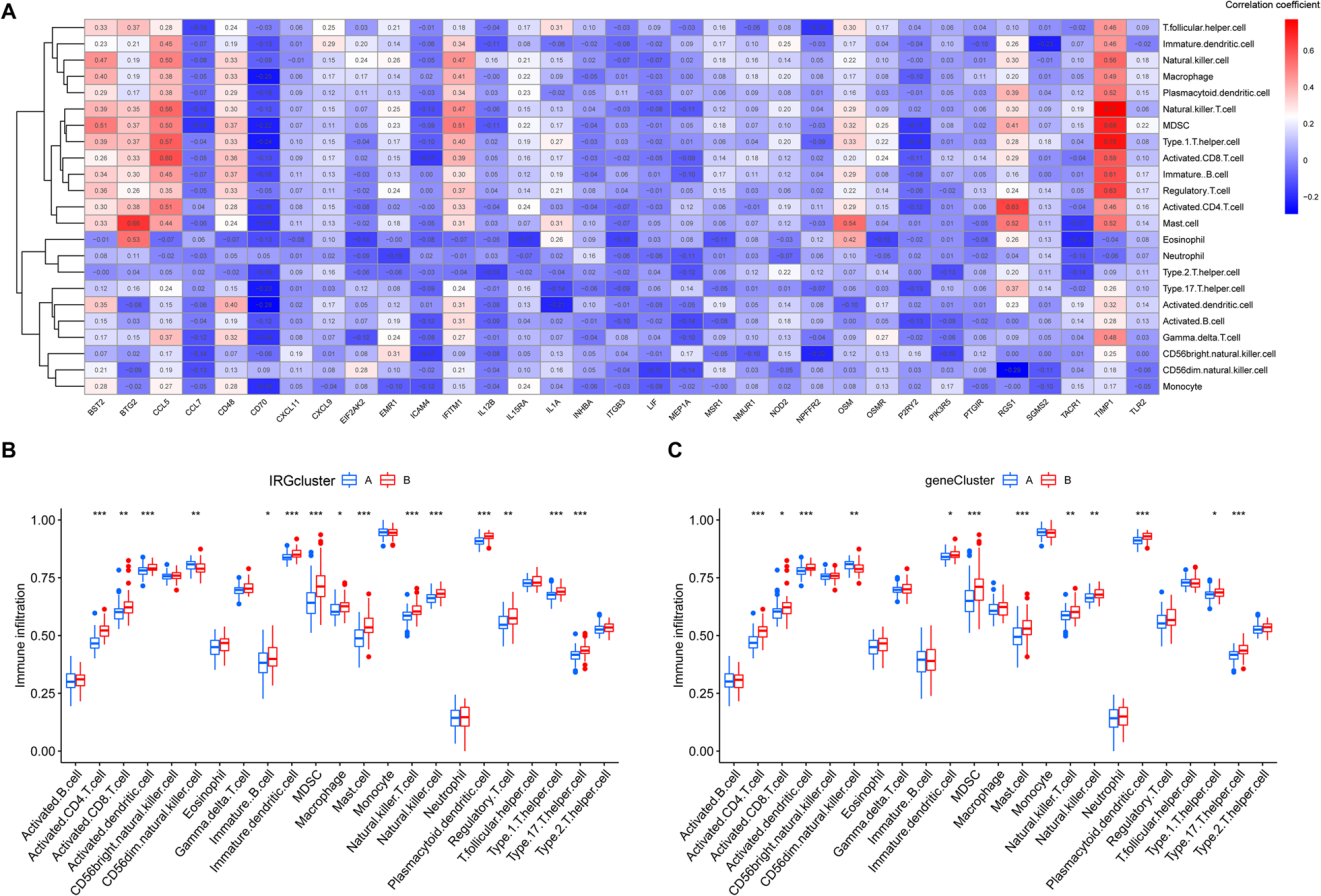
Immune cells infiltration. **A** The correlation between the 33 differentially expressed IRGs and 23 immune cell subtypes. **B** Differential immune cells infiltration in the IRGcluster A group and IRGcluster B group. **C** Differential immune cells infiltration in the genecluster A group and genecluster B group. *p < 0.05, **p < 0.01, ***p < 0.001

**Fig. 8:**
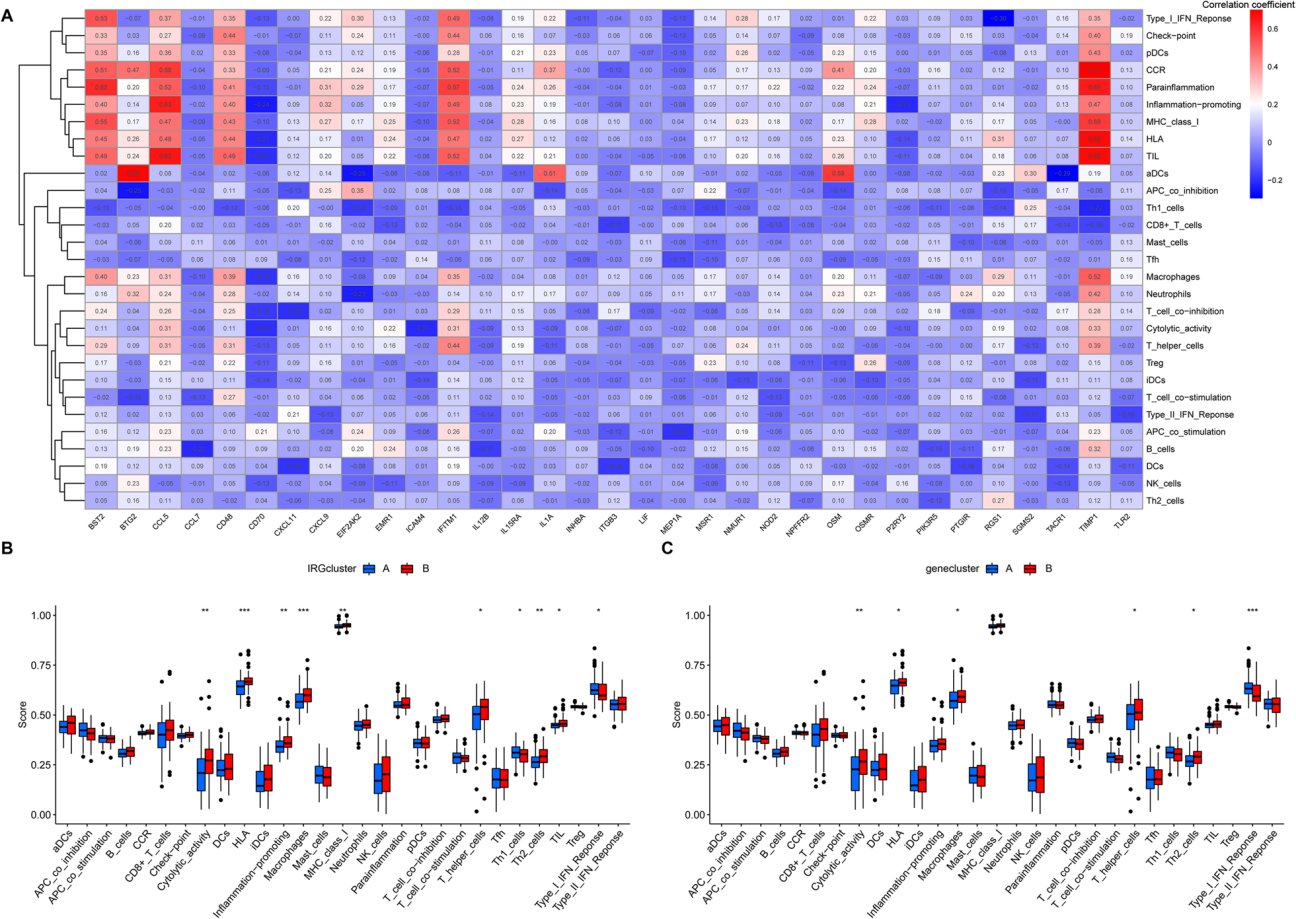
Immune function. **A** The correlation between the 33 differentially expressed IRGs and immune function. **B** Differential immune function in the IRGcluster A group and IRGcluster B group. **C** Differential immune function in the genecluster A group and genecluster B group. *p < 0.05, **p < 0.01, ***p < 0.001

The abundances of 23 immune cell subtypes were determined in TLE patients (Additional file 2: Table S2). We evaluated the correlation between the 33 differentially expressed IRGs and 23 immune cell subtypes and found that TIMP1 was correlated with many types of immune cells, particularly natural killer T-cells and type 1 T helper cells (Fig. 7A). We compared the differential immune cells in the IRGcluster (Fig. 7B) and genecluster subgroups (Fig. 7C), and some immune cell subtypes showed differing infiltration in the IRGcluster and genecluster subgroups. Similarly, we evaluated the correlation between the 33 differentially expressed IRGs and immune function score and found that TIMP1 was correlated with many types of immune function, particularly CCR and parainflammation (Fig. 8A). We also compared the differential immune function in the IRGcluster (Fig. 8B) and genecluster subgroups (Fig. 8C), and some immune function was different in the IRGcluster and genecluster subgroups.

### The correlation between the IRGscore and subtype classification

**Fig. 9:**
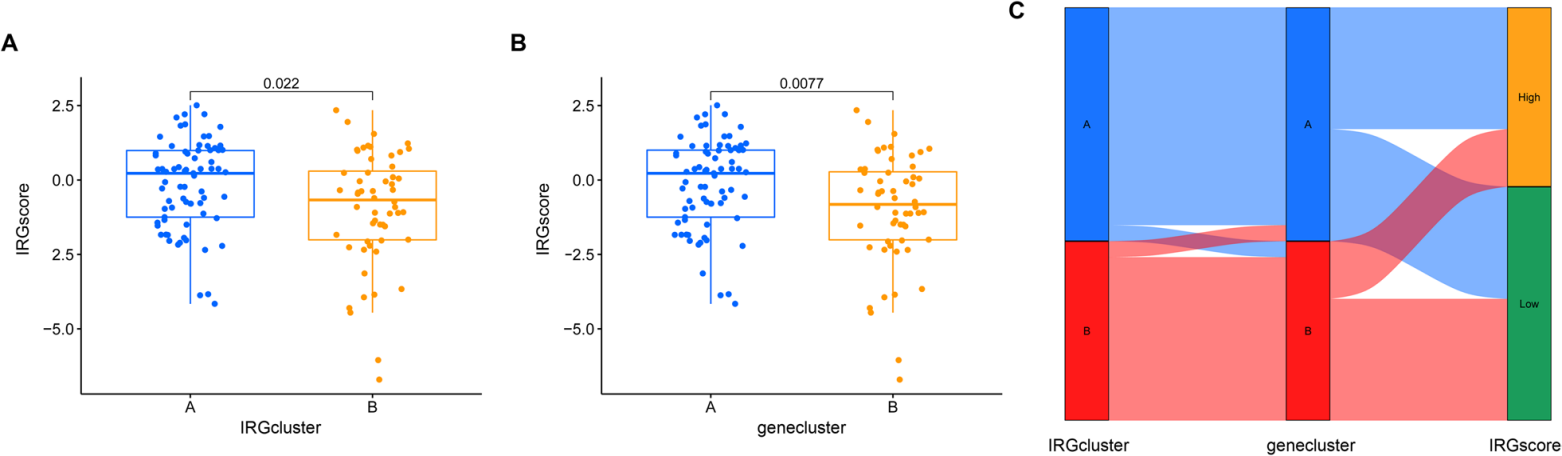
The correlation between IRGscore and subtype classification. **A** Boxplot of the IRGscore in the IRGcluster A group and IRGcluster B group. **B** Boxplot of the IRGscore in the genecluster A group and genecluster B group. **C** Sankey diagram showing the relationship between IRGcluster subgroups, genecluster subgroups and IRGscore

PCA algorithms were utilized to calculate the IRGscore for each TLE patient according to the expression of 33 differentially expressed IRGs. We compared the IRGscore in the IRGcluster (Fig. 9A) and genecluster subgroups (Fig. 9B) and found that TLE patients in the IRGcluster A and genecluster A groups had higher IRGscore values. Moreover, the relationship between IRGcluster subgroups, genecluster subgroups and the IRGscore was visualized in a Sankey diagram (Fig. 9C).

### TIMP1 was associated with immune cell infiltration and immune function

**Fig. 10:**
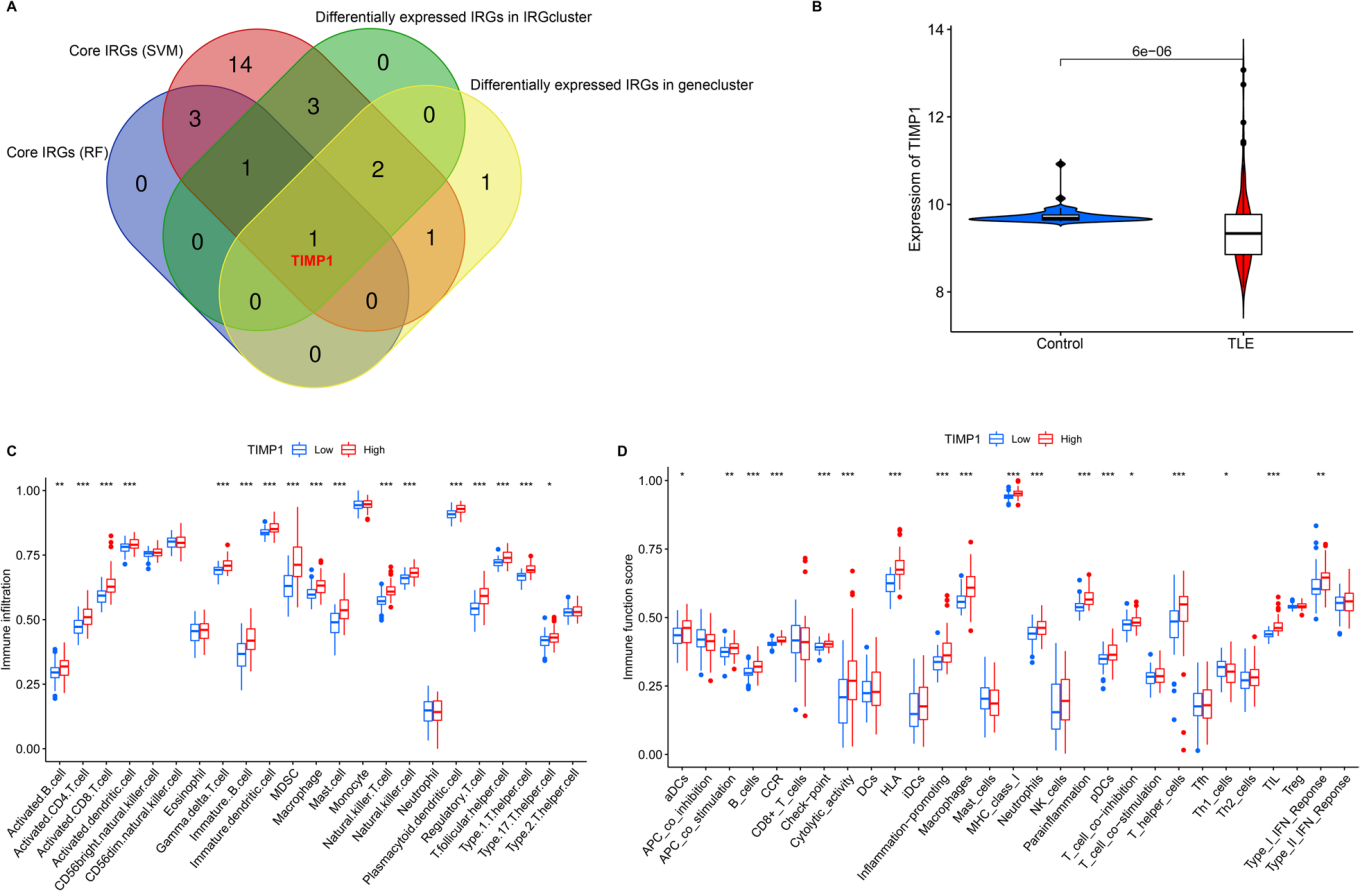
TIMP1 was associated with immune cells infiltration and immune function. **A** Venn diagram based on 33 differentially expressed IRGs in the RF, SVM, IRGcluster and genecluster. **B** Violin plot of TIMP1 expression in the control group and TLE group. **C** Differential immune cell in the TIMP1 low group and TIMP1 high group. **D** Differential immune function in the TIMP1 low group and TIMP1 high group. *p < 0.05, **p < 0.01, ***p < 0.001

Based on 33 differentially expressed IRGs in RF, SVM, IRGcluster and genecluster analyses, we found that TIMP1 was the most significant IRG associated with TLE, and it might be a potential diagnostic biomarker for TLE (Fig. 10A). We compared the expression of TIMP1 in the control group and TLE group and found that its expression was decreased in the TLE group (Fig. 10B). TLE patients were divided into a low group and high group according to the expression of TIMP1. We compared the differential immune cells in the low and high groups and found that TLE patients had higher immune cell infiltration in the TIMP1 low group than in the TIMP1 high group (Fig. 10C). Moreover, we compared the immune function score in the low and high groups and found that TLE patients had higher immune function scores in the TIMP1 low group than in the TIMP1 high group (Fig. 10D).

### The location and expression of TIMP1 in TLE patients and KA-induced epileptic mice

**Fig. 11:**
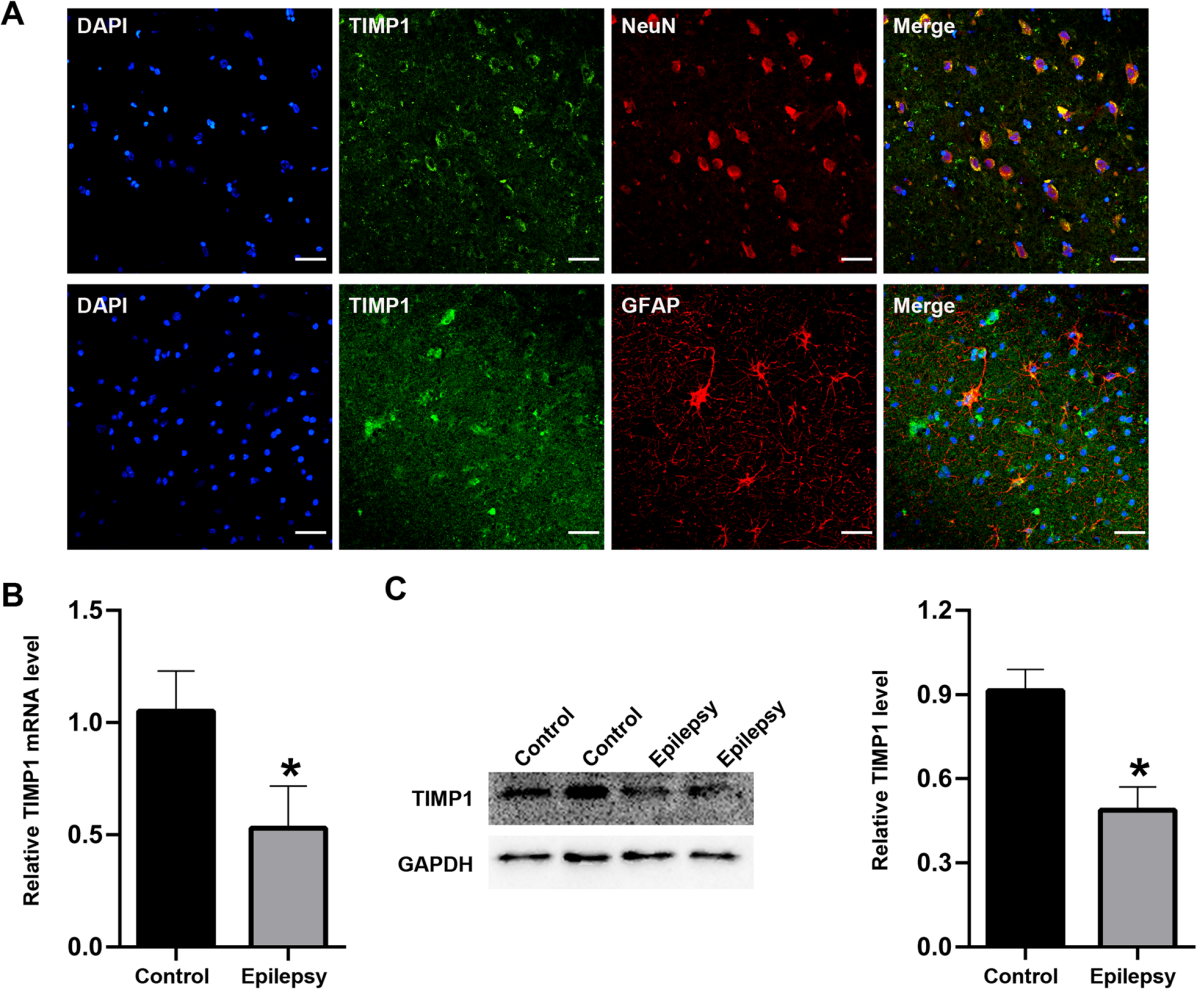
The location and expression of TIMP1 in the TLE patients. **A** Immunofluorescence staining for TIMP1 in the TLE patients (scar bar: 50 μm). **B** qrtPCR for TIMP1 in the controls and TLE patients (*n* = 12/per group). **C** Western blotting for TIMP1 in the controls and TLE patients (*n* = 12/per group). *p < 0.05

**Fig. 12:**
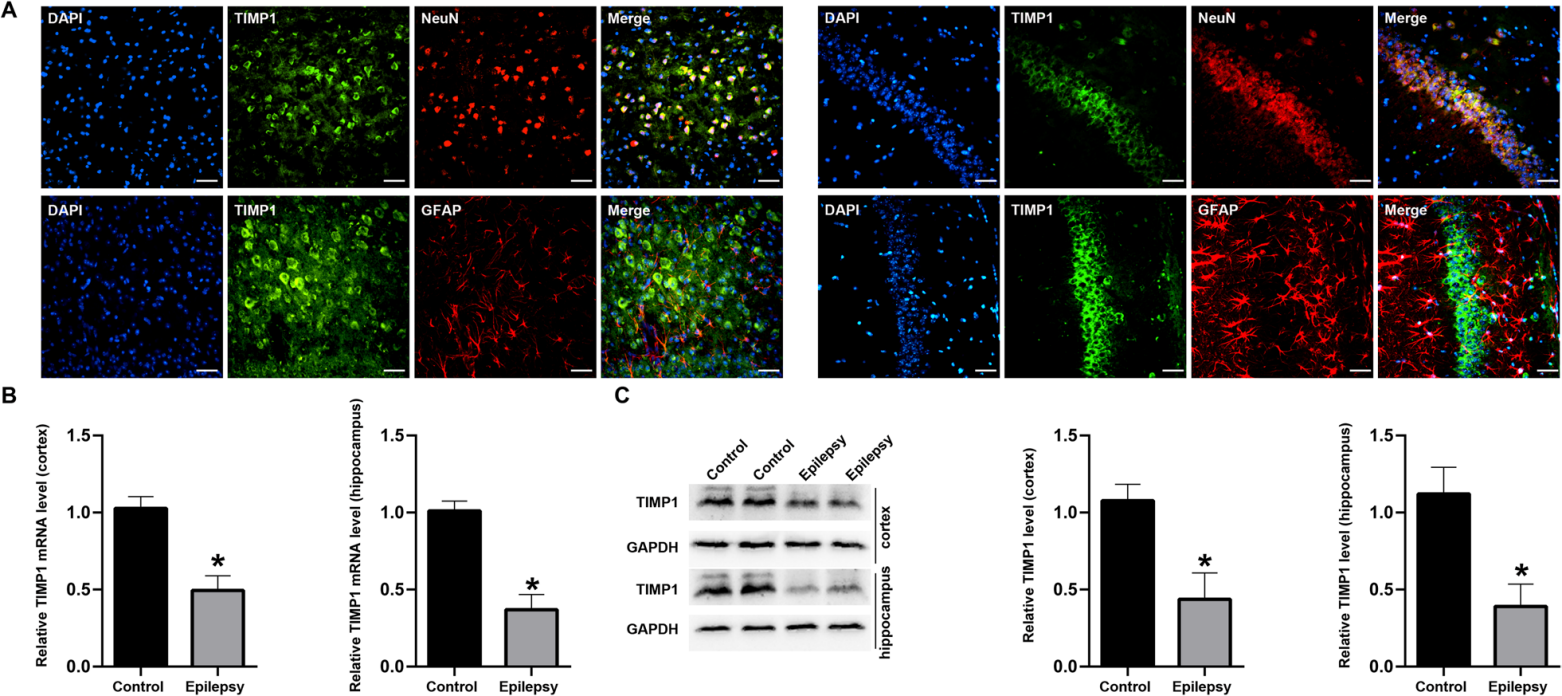
The location and expression of TIMP1 in the KA-induced epilepsy mice. **A** Immunofluorescence staining for TIMP1 in the cortices and hippocampi (CA1 region) of KA-induced epilepsy mice (scar bar: 50 μm). **B** qrtPCR for TIMP1 in the cortices and hippocampi of KA-induced epilepsy mice (*n* = 6/per group). **C** Western blotting for TIMP1 in the cortices and hippocampi of KA-induced epilepsy mice (*n* = 6/per group). *p < 0.05

To further verify the location and expression of TIMP1 in TLE patients and KA-induced epileptic mice, immunofluorescence staining, qRT‒PCR, and western blotting were performed. We found that TIMP1 was mainly located in cortical neurons and scantly expressed in cortical gliocytes (Fig. 11A). qRT‒PCR (Fig. 11B) and western blotting (Fig. 11C) indicated that TIMP1 was decreased in the cortices of TLE patients compared with controls. Similarly, in the cortices and hippocampi (CA1 region) of KA-induced epileptic mice, TIMP1 was mainly located in cortical neurons and was scantly expressed in cortical gliocytes (Fig. 12A). Moreover, qRT‒PCR (Fig. 12B) and western blotting (Fig. 12C) indicated that TIMP1 was decreased in the cortices and hippocampi of KA-induced epilepsy mice compared with controls.

## Discussion

Accumulating evidence strongly indicates the relevance of inflammation in the pathophysiology of epilepsy, as inflammatory mediators are upregulated and neural inflammation is promoted, which may induce neurological pathology changes and contribute to the development of epilepsy [22, 23]. Research on epileptic patients revealed high expression of several pro-inflammatory cytokines in the serum and cerebrospinal fluid [24]. Lines reported that the activation of NLRP1 and NLRP3 inflammasomes is involved in the development of epilepsy [25, 26]. Administration of inhibitors of the inflammasome complex such as MCC950, Ac-YVAD-cmk, CY-09, and Bay11-7082 or other drugs via small interfering RNAs protected the symptoms of epilepsy [27–30]. Neuroinflammation is usually considered to be associated with dysfunction of the BBB, and the infiltration of peripheral immune cells across the BBB has been verified in epileptic patients [31]. However, the specific mechanisms of inflammation in epilepsy need to be further investigated. Therefore, we attempted to elucidate the specific role of IRGs in TLE and the immune microenvironment. Additionally, gene signatures related to inflammation were utilized to predict TLE.

Bioinformatics analysis plays an important role in the diagnosis and prognosis of diseases, and it promotes the understanding of disease processes via genome-level and systematic bioinformatics methods. In our research, we filtered 33 differentially expressed IRGs by comparing the expression of IRGs in the hippocampi of controls and TLE patients. Then, cluster analyses were performed in TLE patients to obtain two groups based on 33 differentially expressed IRGs, and 48 IRG-related DEGs were identified. Then, those 48 IRG-related DEGs were screened for GO, KEGG and GSEA enrichment analysis. The results showed that IRG-related DEGs were significantly enriched in inflammation-related pathways and immune-related pathways, such as the MAPK cascade, IL-17 signaling pathway, NF-κB signaling pathway, and chemokine binding. A previous study found an expansion of the CD4 T-cell subset in the peripheral blood and a shift toward a proinflammatory Th17/Th1 CD4 T-cell immune profile in drug-resistant epilepsy [9]. Activation of the NF-κB signaling pathway can increase the expression of proinflammatory cytokines and trigger a signaling cascade, contributing to oxidative stress, neurogenesis, neuronal death survival, and synaptic plasticity [32]. To validate the accuracy of inflammation clustering, we performed a second unsupervised gene consensus cluster based on 33 differentially expressed IRGs and 48 IRG-related DEGs, and the TLE patients were divided into two IRG clusters and two gene clusters. IRGcluster B had high expression of IRGs, including BST2, BTG2, CCL5, IFITM1, OSM, RGS1 and TIMP1. Genecluster B had high expression of IRGs, including BTG2, IL1A, RGS1, TIMP1 and TLR2. Regarding the immune microenvironment, both IRGcluster B and genecluster B had increased immune scores, immune infiltration, and immune function, including increases in CD4 T-cells, CD8 T-cells, dendritic cells, myeloid-derived suppressor cells (MDSCs), and cytolytic activity. Pitsch et al. demonstrated spontaneous recurrent seizures and persisting memory deficits, and the sclerotic hippocampus was populated with CD8^+^ T-cells escorted by NK-cells [33]. MDSCs can inhibit other immune cells, including T-, B-, and NK-cells, and are closely related to many nervous system diseases [34, 35]. Taken together, differentially expressed IRGs and IRG-related DEGs were likely associated with neuroinflammation and the immune microenvironment.

Based on the 33 differentially expressed IRGs, we identified TIMP1 as the most significant IRG associated with TLE according to RF, SVM, nomogram, subtype classification, enrichment, PPI, immune cell infiltration, and immune function analyses. We found that TIMP1 was decreased in TLE patients and was associated with immune infiltration and immune function. The patients with higher TIMP1 expression had more immune cell infiltration and stronger immune function. Moreover, in TLE patients and KA-induced epileptic mice, immunofluorescence staining indicated that TIMP1 was mainly located in neurons and expressed at low levels in gliocytes. qRT‒PCR and western blotting verified that TIMP1 was decreased in TLE patients and KA-induced epileptic mice compared with their controls. TIMP1 inhibits matrix metallopeptidase-9 (MMP-9), which is a major component of the basement membrane of the cerebral endothelium, and both are key regulators of inflammation [36]. MMP-9 degrades collagen IV and promotes the migration of cells through tissues and across the BBB. The MMP-9/TIMP1 ratio may reflect the state of the BBB. A high MMP-9/TIMP1 ratio in pediatric patients is closely tied to encephalitis and prolonged febrile seizures [37]. These results implied that the MMP-9/TIMP1 ratio might be associated with inflammation and dysfunction of the BBB [38, 39]. The microvasculature in the BBB is crucial for the maintenance of brain homeostasis, and research has reported that BBB microvascular malfunction induces epilepsy. Tinnes et al. proved that epileptic conditions strongly induce TIMP1 synthesis in the hippocampus, which in turn blocks MMP activity to protect the BBB, prevent inflammatory cytokine overflow, and weaken epilepsy, indicating the protective effect of TIMP1, which is consistent with the results of our study that the expression of TIMP1 was decreased in epileptic patients and mice [40]. Moreover, BBB permeability also plays a key role in the development and progression of epilepsy, while increased BBB permeability may induce the effusion of proinflammatory cytokines into the brain [41, 42].

However, our study had several limitations. First, in addition to TIMP1 as the core IRG in the diagnosis of TLE, other IRGs, such as BST2, BTG2, CCL5, IFITM1, OSM, and RGS1, might also be potential diagnostic biomarkers for TLE, and these IRGs require further studies. For example, Pawel Wolinski et al. observed the upregulation of IL1β and CXCL12 in the early phase of KA-induced epilepsy and elevated levels of CCL5 at a later time point, indicating the important roles of these IRGs in epilepsy [43]. Second, larger and more diverse samples are needed to provide a broader understanding of the heterogeneity of TLE and strengthen the conclusions. Furthermore, based on previously published studies, we believe that TIMP1 can not only directly mediate inflammation-related pathways but also indirectly modulate the entry of inflammatory factors into the brain by regulating the permeability of the BBB, and these pathological processes are closely related to the development and progression of epilepsy, which also requires further study. Finally, studies on other independent datasets and next-generation sequencing datasets as well as across different research centers are needed to provide results with greater reliability and robustness.

In conclusion, we identified TIMP1 as the most significant IRG associated with epilepsy and found the downregulated expression of TIMP1 in epileptic patients and mice, which may provide new ideas for studying the mechanism of epilepsy and discovering new drugs for treatment.

## Supplementary Information

**Additional file 1: Table S1.** Differentially expressed genes in the IRGcluster A group and IRGcluster B group.

**Additional file 2: Table S2.** The abundance of 23 immune cell subtypes in TLE patients.

## Data Availability

Data of this study could be accessed freely under reasonable request.

## References

[1] Devinsky O, Vezzani A, O'Brien TJ, Jette N, Scheffer IE (2018). Epilepsy. Nat Rev Dis Prim.

[2] Godeau D, Petit A, Richard I, Roquelaure Y, Descatha A (2021). Return-to-work, disabilities and occupational health in the age of COVID-19. Scand J Work Environ Health.

[3] Téllez-Zenteno JF, Hernández-Ronquillo L (2012). A review of the epidemiology of temporal lobe epilepsy. Epilepsy Res Treat.

[4] Paudel YN, Shaikh MF, Shah S, Kumari Y, Othman I (2018). Role of inflammation in epilepsy and neurobehavioral comorbidities: implication for therapy. Eur J Pharmacol.

[5] Perucca E, Brodie MJ, Kwan P, Tomson T (2020). 30 years of second-generation antiseizure medications: impact and future perspectives. Lancet Neurol.

[6] Vezzani A, Aronica E, Mazarati A, Pittman QJ (2013). Epilepsy and brain inflammation. Exp Neurol.

[7] Jiang NM, Cowan M, Moonah SN, Petri WA (2018). The impact of systemic inflammation on neurodevelopment. Trends Mol Med.

[8] Dong X, Fan J, Lin D, Wang X, Kuang H, Gong L (2022). Captopril alleviates epilepsy and cognitive impairment by attenuation of C3-mediated inflammation and synaptic phagocytosis. J Neuroinflamm.

[9] Kamali AN, Zian Z, Bautista JM, Hamedifar H, Hossein-Khannazer N, Hosseinzadeh R (2021). The potential role of pro-inflammatory and anti-inflammatory cytokines in epilepsy pathogenesis. Endocr Metab Immune Disord Drug Targets.

[10] Vezzani A, Balosso S, Ravizza T (2008). The role of cytokines in the pathophysiology of epilepsy. Brain Behav Immun.

[11] Stellwagen D, Beattie EC, Seo JY, Malenka RC (2005). Differential regulation of AMPA receptor and GABA receptor trafficking by tumor necrosis factor-alpha. J Neurosci.

[12] Miller JW (2021). Inflammation as a target for epilepsy therapy: the case of natalizumab. Neurology.

[13] Rigatti SJ (2017). Random forest. J Insur Med.

[14] Huang S, Cai N, Pacheco PP, Narrandes S, Wang Y, Xu W (2018). Applications of support vector machine (SVM) learning in cancer genomics. Cancer Genom Proteom.

[15] Robin X, Turck N, Hainard A, Tiberti N, Lisacek F, Sanchez JC (2011). pROC: an open-source package for R and S+ to analyze and compare ROC curves. BMC Bioinform.

[16] Iasonos A, Schrag D, Raj GV, Panageas KS (2008). How to build and interpret a nomogram for cancer prognosis. J Clin Oncol.

[17] Wilkerson MD, Hayes DN (2010). ConsensusClusterPlus: a class discovery tool with confidence assessments and item tracking. Bioinformatics.

[18] Dai B, Sun F, Cai X, Li C, Liu H, Shang Y (2021). Significance of RNA N6-methyladenosine regulators in the diagnosis and subtype classification of childhood asthma using the gene expression omnibus database. Front Genet.

[19] Bindea G, Mlecnik B, Tosolini M, Kirilovsky A, Waldner M, Obenauf AC (2013). Spatiotemporal dynamics of intratumoral immune cells reveal the immune landscape in human cancer. Immunity.

[20] Liu Y, Wang Y, Yang J, Xu T, Tan C, Zhang P (2022). G-alpha interacting protein interacting protein, C terminus 1 regulates epileptogenesis by increasing the expression of metabotropic glutamate receptor 7. CNS Neurosci Ther.

[21] Liu Y, Wang T, Liu X, Wei X, Xu T, Yin M (2017). Neuronal zinc-α2-glycoprotein is decreased in temporal lobe epilepsy in patients and rats. Neuroscience.

[22] Vezzani A, Maroso M, Balosso S, Sanchez MA, Bartfai T (2011). IL-1 receptor/Toll-like receptor signaling in infection, inflammation, stress and neurodegeneration couples hyperexcitability and seizures. Brain Behav Immun.

[23] Cerri C, Caleo M, Bozzi Y (2017). Chemokines as new inflammatory players in the pathogenesis of epilepsy. Epilepsy Res.

[24] Aronica E, Crino PB (2011). Inflammation in epilepsy: clinical observations. Epilepsia.

[25] Cristinade Brito Toscano E, Leandro MVÉ, Boni Rocha Dias B, VidigalCaliari M, Paula Gonçalves A, Varela Giannetti A (2021). NLRP3 and NLRP1 inflammasomes are up-regulated in patients with mesial temporal lobe epilepsy and may contribute to overexpression of caspase-1 and IL-β in sclerotic hippocampi. Brain Res.

[26] Mohseni-Moghaddam P, Roghani M, Khaleghzadeh-Ahangar H, Sadr SS, Sala C (2021). A literature overview on epilepsy and inflammasome activation. Brain Res Bull.

[27] Amorim RP, Araújo M, Valero J, Lopes-Cendes I, Pascoal V, Malva JO (2017). Silencing of P2X7R by RNA interference in the hippocampus can attenuate morphological and behavioral impact of pilocarpine-induced epilepsy. Purinergic Signal.

[28] Rao MS, Hattiangady B, Rai KS, Shetty AK (2007). Strategies for promoting anti-seizure effects of hippocampal fetal cells grafted into the hippocampus of rats exhibiting chronic temporal lobe epilepsy. Neurobiol Dis.

[29] Shen K, Jiang W, Zhang C, Cai L, Wang Q, Yu H (2021). Molecular mechanism of a specific NLRP3 inhibitor to alleviate seizure severity induced by pentylenetetrazole. Curr Mol Pharmacol.

[30] Wu Q, Wang H, Liu X, Zhao Y, Zhang J (2022). The role of the negative regulation of microglia-mediated neuroinflammation in improving emotional behavior after epileptic seizures. Front Neurol.

[31] Vezzani A, Friedman A, Dingledine RJ (2013). The role of inflammation in epileptogenesis. Neuropharmacology.

[32] Cai M, Lin W (2022). The function of NF-Kappa B during epilepsy, a potential therapeutic target. Front Neurosci.

[33] Pitsch J, van Loo K, Gallus M, Dik A, Kamalizade D, Baumgart AK (2021). CD8(+) T-lymphocyte-driven limbic encephalitis results in temporal lobe epilepsy. Ann Neurol.

[34] Salminen A, Kaarniranta K, Kauppinen A (2018). The potential importance of myeloid-derived suppressor cells (MDSCs) in the pathogenesis of Alzheimer’s disease. Cell Mol Life Sci.

[35] Sribnick EA, Popovich PG, Hall MW (2022). Central nervous system injury-induced immune suppression. Neurosurg Focus.

[36] Sharma C, Dobson GP, Davenport LM, Morris JL, Letson HL (2021). The role of matrix metalloproteinase-9 and its inhibitor TIMP-1 in burn injury: a systematic review. Int J Burns Trauma.

[37] Palus M, Zampachová E, Elsterová J, Růžek D (2014). Serum matrix metalloproteinase-9 and tissue inhibitor of metalloproteinase-1 levels in patients with tick-borne encephalitis. J Infect.

[38] Matsuura R, Hamano SI, Daida A, Nonoyama H, Kubota J, Ikemoto S (2020). Serum matrix metallopeptidase-9 and tissue inhibitor of metalloproteinase-1 levels in autoimmune encephalitis. Brain Dev.

[39] Ichiyama T, Takahashi Y, Matsushige T, Kajimoto M, Fukunaga S, Furukawa S (2009). Serum matrix metalloproteinase-9 and tissue inhibitor of metalloproteinase-1 levels in non-herpetic acute limbic encephalitis. J Neurol.

[40] Tinnes S, Ringwald J, Haas CA (2013). TIMP-1 inhibits the proteolytic processing of Reelin in experimental epilepsy. FASEB J.

[41] Vishwakarma S, Singh S, Singh TG (2022). Pharmacological modulation of cytokines correlating neuroinflammatory cascades in epileptogenesis. Mol Biol Rep.

[42] Bankstahl M, Breuer H, Leiter I, Märkel M, Bascuñana P, Michalski D (2018). Blood-brain barrier leakage during early epileptogenesis is associated with rapid remodeling of the neurovascular unit. eNeuro..

[43] Wolinski P, Ksiazek-Winiarek D, Glabinski A (2022). Cytokines and neurodegeneration in epileptogenesis. Brain Sci.

